# Applied Chemistry

**Published:** 1869-02-01

**Authors:** 

**Affiliations:** From the Proceedings of the Biological Society, Paris


### *Applied Chemistry.*

*Application of the Atomic or Thermic Law to Biatomic Metalloids. By Dr. Rabuteau. From the Proceedings of the Biological Society, Paris. Report of Gazette Mèdicale.*

TRANSLATED FOR THIS JOURNAL, BY W. HAY, M. D., ASSOCIATE EDITOR.

I discovered, last year, a law, to the demonstration of which I devoted the greater portion of my inaugural thesis (*Vide* Experimental Investigations into the Physiological Effects of Fluorides, and of Metallic Compounds in General. Paris: Germer Ballière, 1867, and Gazette Hebdomadaire, 15 May, 1868).

According to this law, the metals are so much the more active as their atomic weight is higher or their specific heat lower; so that two or more metals being given, whatever may be their chemical analogies, their relative activities may be determined according to their atomic weights or their specific heat. Let us take, for example, sodium (atomic weight, 39), thallium (atomic weight, 204); the sodium is inoffensive, the potassium is a muscular poison in large doses, and the thallium is as terrible as lead.

When I speak of metals, it is, of course, to be understood that I speak of their salts. Compare zinc and cadmium, whose chemical analogies are remarkable, and it will be seen that the cadmium, whose atomic weight is 112, is much more active than the zinc, having an atomic weight of 62.02. I believe I am warranted in the assertion that iridium, whose atomic weight is 71.08, will present physiological properties (relations?) intermediate as to their energy, between those of zinc and cadmium. I believed that the atomic law should be applied to a certain number of metalloids. The first group of these simple bodies constitutes, as is well known, an exception to the law of Bouchardat and Stuart Cooper; but must the same be true of the other groups? I then examined the biatomic metalloids and discovered a new and remarkable application of the atomic law. The metalloids which form the second group are the following:

| | | |
|---|---|---|
| Oxygen, atomic weight | | 16.00 |
| Sulphur, " | " | 32.00 |
| Selenium, " | " | 79.50 |
| Tellurium, " | " | 128.28 |

The chemical properties of these simple bodies, especially of the three last, present the greater analogies, so that they have always been associated in the same natural group. But in this connection, however, when it concerns biatomic metals, it is necessary to appreciate the physical properties which the atoms possess, the property of heat and consequently that of motion, for life is only motion. If the hydrogen compounds of these metalloids are considered, it will be seen that water $H^2O$ is indispensable to life.

That sulphuretted hydrogen $H^2S$ is a deleterious gas, but that nevertheless it may be introduced in considerable quantity into the digestive tube, into the venous blood (Cl. Bernard), whilst seleniuretted hydrogen $H^2Se$. is exceedingly dangerous. Berzelius nearly perished from having inspired only a very small quantity, and all those engaged in the examination of this gas know that a single bubble introduced into the nasal fossæ, produces coryza, sneezing and the abolition of the sense of smell during several days. As to tellurietted hydrogen $H^2Te$. an unstable gas very little examined hitherto, it should possess toxic properties in the very highest degree. I propose to experiment by causing animals to inhale atmospheric air charged with certain proportions of these three last named gases.

The same relative differences in physiological energy are recogized if the oxygen compounds of sulphur, selenium and tellurium are compared together. The sulphites, hyposulphites, sulphates and hyposulphates may be said to be inoffensive, if the metal they contain has but little activity. This was already well understood of the first classes of the preceding salts. I have demonstrated that the hyposulphates come under the general rule, that the hyposulphate of soda for example may be taken in considerable doses, for I have injected five grammes of this salt into the veins of a dog without producing any alteration in the health of the animal. Moreover I myself took five grammes of the same salt, without experiencing any other than a very slight laxative effect. The selenites and the seleniates are on the contrary eminently toxic; they rapidly destroy animals by asphyxia with dilation of the pupils and of all the sphincters. Finally the tellurites, from a single experiment which I made appeared to me to be still more terrible, for after having injected, into the veins of a medium sized dog, a very feeble dose of tellurite of soda, this salt produced the gravest disorders which I had ever seen.

To render more apparent the differences of action of the compounds which I have examined, I will tabulate the principal circumstances of my experiments and their results, viz:

| Animal. | Salt. | Dose injected into the veins. | Dose carried into the stomach. | Result. |
|---|---|---|---|---|
| | | Grammes. | Grammes. | |
| Bitch...... | Sulphate of Soda................. | 7 | " | Negative. |
| Dog......... | id ............ | 14 | " | id |
| Bitch...... | Sulphite of Soda................. | 4.78 | " | id |
| Dog......... | Hyposulphite of Soda........ | 4 | " | id |
| Bitch...... | Selenite of Soda.................. | 0.25 | " | Death. |
| Dog......... | id ............ | 0.12 | " | id |
| Rabbit... | id ,............ | 0.12 | 0,50 | id |
| Dog......... | id ............ | 0.12 | 0.80 | Transient effect. |
| Bitch,..... | Selenite of Copper ............ | 0.12 | 0.50 | id |
| Dog......... | Seleniate of Potassa.......... | 0.25 | 0.50 | Death. |
| Dog......... | Tellurite of Soda............... | 0.08 | 0.50 | Death. |

I propose soon to acquaint you with the toxic effects of the compounds of selenium and tellurium. I will only say that I classify these amongst the mechanical poisons, to which class I assign a different definition from that which has hitherto been given to them. Indeed, I do not admit, as mechanical poisons, glass, diamonds, the nails, &c., for these are only foreign bodies. In my opinion a mechanical poison is a substance which, introduced into the interior of the system, produces death by creating mechanical obstacles to the accomplishment of one or more of its functions. Inasmuch as these obstacles result from the metamorphoses of the substance which has penetrated into the organism, by so much do they originate from them, and their production is provoked by the poison. It is thus that the selenites induce death in the midst of frightful asphyxia, and the blood is filled with a multitude of little crystals, sometimes as numerous as the globules. I am still ignorant of the nature of these crystals.

I have already developed my ideas upon the subject of mechanical poisons in a public course of toxicology, delivered at the School of Practice. I stated why I admitted three classes of poisons, viz: 1st, mechanical poisons; 2d, caustics or corrosive poisons; 3d, biological poisons. The tellurites and tellurates, as well as the seleniates belong to the first class.

As to the metamorphoses of selenites and seleniates, I can assert already that the compounds undergo a reduetion in the organism. Indeed, having injected twenty-five centigrammes of seleniate of potassa into the veins of a dog, the breath of this animal, some hours after the injection, exhaled a strong odor of seleniuretted hydrogen. The oxygen compounds of tellurium would appear to undergo likewise a reduction in the organism. Indeed, Gonelin (*) having tried telluric acid on rabbits, states that the mucus and the feces were colored black by the reduced tellurium.

### *Conclusion of Unpublished Investigations.*

BY DR. RABUTEAU.

1st. The *chlorate of potassa* is totally eliminated in its natural state. I believe that I am the first who has demonstrated this fact experimentally, by absolving chlorate of potassa and making a *quantitutive analysis* of the salt recovered in the urine. Möhler, as early as 1824, and subse-

(*) Husemann, *Handbuch der Toxicologie.*

